# Focused Ultrasound Surveillance of Lymph Nodes Following Lymphoscintigraphy Without Sentinel Node Biopsy: A Useful and Safe Strategy in Elderly or Frail Melanoma Patients

**DOI:** 10.1245/s10434-019-07505-6

**Published:** 2019-06-25

**Authors:** Norbertus A. Ipenburg, John F. Thompson, Roger F. Uren, David Chung, Omgo E. Nieweg

**Affiliations:** 1 Department of Dermatology, Leiden University Medical Center (LUMC), Leiden, Netherlands; 2 Melanoma Institute Australia, Wollstonecraft, North Sydney, NSW Australia; 3 Sydney Medical School, University of Sydney, Sydney, NSW Australia; 4 Department of Melanoma and Surgical Oncology, Royal Prince Alfred Hospital, Sydney, NSW Australia; 5 Alfred Nuclear Medicine and Ultrasound, RPAH Medical Centre, Sydney, NSW Australia

## Abstract

**Background:**

Sentinel node (SN) biopsy (SNB) has become standard of care in clinically localized melanoma patients. Although it is minimally invasive, advanced age and/or comorbidities may render SNB inadvisable in some patients. Focused ultrasound follow-up of SNs identified by preoperative lymphoscintigraphy may be an alternative in these patients. This study examines the outcomes in patients managed in this way at a major melanoma treatment center.

**Methods:**

All patients with clinically localized cutaneous melanoma who underwent lymphoscintigraphy and in whom SNB was intentionally not performed due to advanced age and/or comorbidities were included.

**Results:**

Between 2000 and 2009, 160 patients (5.2% of the total) underwent lymphoscintigraphy without SNB because of advanced age and/or comorbidities. Compared with the 2945 patients who had a SNB, the 160 patients were older, had thicker melanomas that were more often located in the head and neck region, and had more SNs in more nodal regions. Of the 160 patients, 150 (94%) were followed with ultrasound examination of their SNs at each follow-up visit; this identified 33% of the nodal recurrences before they became clinically apparent. Compared with SN-positive patients who were treated by completion lymph node dissection, observed patients who developed nodal recurrence had more involved nodes when a delayed lymphadenectomy was performed. Melanoma-specific survival, recurrence-free survival, and distant recurrence-free survival rates were similar, while regional lymph node-free survival was worse.

**Conclusions:**

Lymphoscintigraphy with focused ultrasound follow-up of SNs is a reasonable management alternative to SNB in patients who are elderly and/or have substantial comorbidities.

Sentinel node (SN) biopsy (SNB) is a routine procedure in patients with clinically localized primary cutaneous melanoma. It offers prognostic and staging information and prolongs survival in SN-positive patients with intermediate-thickness melanomas.^1^,^2^ Recent studies have demonstrated that adjuvant immunotherapy and targeted therapy improve survival of stage III patients, including those with minimal nodal involvement. These findings further increase the significance of SNB.^3^–^5^ However, the procedure may be considered inappropriate in some patients, for various reasons. Elderly patients, for example, have a significantly reduced risk of nodal involvement and a higher risk of complications.^6^–^8^ The drawbacks may also outweigh the benefits in patients with substantial comorbidities. At Melanoma Institute Australia (MIA), SNB is sometimes intentionally avoided in such patients. Lymphoscintigraphy is still performed and the location of each SN is marked on the overlying skin with a minute tattoo spot. These nodes are then examined and followed with focused high-resolution ultrasonography (US). This strategy is not known to be practiced elsewhere on a regular basis.

The aim of this study was to assess our experience with this approach. Specific matters to be assessed were the prevalence of omitting the SNB, the reason(s), characteristics of these patients, the follow-up strategy, the stage of the disease at the time of a regional node field recurrence, and the ways in which these metastases were detected and managed. Survival was compared with that of patients who did undergo SNB.

## Patients and Methods

### Patients

The database of MIA, which contains prospectively collected information, was queried for all patients with clinically localized cutaneous melanoma who underwent SNB between November 2000 and December 2009 (SNB patients) and all patients in whom lymphoscintigraphy and US were performed but in whom SNB was intentionally not scheduled due to advanced age and/or comorbidities (observed patients). Patients were excluded if they had melanoma in situ, multiple primary melanomas (micro)satellites or in-transit metastases, if preoperative ultrasound revealed nodal metastasis, if no SN was identified intraoperatively, if wide local excision had been performed before lymphoscintigraphy, or if SNB had been performed elsewhere. The study was approved by the MIA Research Committee. Written informed consent was obtained from all patients.

### Lymphoscintigraphy and Sentinel Node Biopsy

A SN was defined as a node on a direct lymphatic drainage pathway from the primary tumor.^9^ SNB was offered to patients with clinically localized melanoma with a Breslow thickness ≥ 1 mm, or for melanomas < 1 mm if adverse features were present, such as young age, ulceration of the primary tumor, Clark level IV or V invasion, or a tumor mitotic rate ≥ 1. Details of the lymphatic mapping and SNB techniques used at MIA have been described previously.^10^ In short, preoperative dynamic and static lymphoscintigraphy were performed. Since 2008, single photon emission computed tomography with integrated computerized tomography (SPECT/CT) has been routinely performed. The location of each SN was marked on the skin with a pin-point permanent tattoo. Patent blue dye and a gamma ray detection probe were used for intraoperative detection of the identified SNs. SNs were serially sectioned and were examined using S100 and HMB-45 immunohistochemistry.^11^ Completion lymph node dissection (CLND) was typically performed in patients with an involved SN, unless they participated in a study (MSLT-II) and were randomized to observation of the nodal region.^12^

### Follow-Up

In patients who were observed, focused high-resolution US of the marked SN basin was performed at each follow-up visit. Lymph nodes were considered to be abnormal if focal low-level internal echoes were present in the cortex of the node or the node had become rounded in shape with the hilum displaced to the side or completely obliterated by low-level internal echoes.^13^ Subcapsular thickening of > 2.5 mm over a section of the node was also considered abnormal. Fine needle aspiration biopsy was performed in patients with nodes that were considered to be suspicious for metastasis on US assessment. Follow-up was every 4 months for the first 2 years, every 6 months for the next 3 years, and annually thereafter.

### Statistical Analysis

Baseline characteristics of patients in the observation and SNB groups were compared. Comparison of continuous variables was performed using the Mann–Whitney *U* test, and values of categorical variables were compared using the Pearson’s Chi-square test or Fisher’s exact test, as appropriate. Melanoma-specific survival (MSS) was calculated from the date of diagnosis to the date of melanoma-related death. Censoring for MSS occurred at the date of death from non-melanoma cause or at the end of follow-up, whichever came first. The event of interest was first recurrence for recurrence-free survival (RFS), first distant recurrence for distant RFS (DRFS), and first regional node recurrence for regional lymph node-free survival (RLNFS). Kaplan–Meier curves were created and covariates were compared using the log-rank test. Type of management was the variable of interest in this study. To adjust for potential confounders, known prognostic factors (sex, age, primary tumor site, Breslow thickness, tumor mitotic rate, and ulceration) were added to the multivariable Cox proportional hazards models.^14^–^18^ To increase the validity of the predictions outside the studied cohort, stepwise methods were not used and full models were built.^19^ The proportional hazards assumption was checked for all included variables. *P* values were two-sided and were considered statistically significant if < 0.05. Statistical analyses were performed using SPSS 25.0 software for Mac (IBM Corporation, Armonk, NY, USA).

## Results

### Cohort Characteristics

Between 2000 and 2009, 2945 patients with clinically localized cutaneous melanoma underwent SNB, and 160 patients (5.2% of the total) underwent lymphoscintigraphy and US, but not SNB because of advanced age and/or comorbidities. Table 1 shows the clinical and pathology characteristics of all patients. Observed patients were older (median 81 vs. 58 years, *p* < 0.001) than SNB patients. The youngest observed patient was 26 years of age and the oldest was 95 years. Fourteen patients (9%) were < 65 years of age. Morbid obesity, cardiovascular disease, pulmonary embolism, schizophrenia, aplastic anemia with thrombocytopenia, penile malignancy with radiotherapy to both groins, pregnancy, rheumatoid arthritis, and wheelchair-bound multiple sclerosis were the reasons for not scheduling the SNB in these patients. Compared with SNB patients, melanomas of observed patients were significantly thicker (median 2.5 vs. 1.8 mm; *p* < 0.001), had a higher tumor mitotic rate (median 4 vs. 3/mm^2^; *p* = 0.002), and were more frequently located in the head and neck region (34% vs. 16%; *p* < 0.001). In observed patients, lymphoscintigraphy revealed drainage to more nodal regions (*p* = 0.004) and more SNs (*p* = 0.04).

**Table 1:** Clinicopathologic characteristics of patients in this study

Characteristic	Observation (*n* = 160)	SNB (*n* = 2945)	*P* value
Sex			0.82^a^
Male	97 (60.6)	1758 (59.7)	
Female	63 (39.4)	1187 (40.3)	
Age (years)			< 0.001^b^
< 65	14 (8.8)	1967 (66.8)	
65–74	18 (11.3)	585 (19.9)	
75–84	81 (50.6)	351 (11.9)	
≥ 85	47 (29.4)	42 (1.4)	
Median (IQR)	81 (76–86)	58 (46.5–69.5)	
Melanoma location			< 0.001^a^
Head and neck	55 (34.4)	465 (15.8)	
Upper limb	48 (30.0)	773 (26.2)	
Lower limb	20 (12.5)	740 (25.1)	
Trunk	37 (23.1)	967 (32.8)	
Breslow thickness, mm			< 0.001^b^
0–1	9 (5.6)	424 (14.4)	
1.01–2	55 (34.4)	1283 (43.6)	
2.01–4	51 (31.9)	836 (28.4)	
> 4	45 (28.1)	394 (13.4)	
Missing	0 (0)	8 (0.3)	
Median (IQR)	2.5 (1.1–3.9)	1.8 (0.95–2.65)	
Tumor mitotic rate (mm^2^)			0.002^b^
0	10 (6.3)	290 (9.8)	
≥ 1	141 (88.1)	2519 (85.5)	
Missing	9 (5.6)	136 (4.6)	
Median (IQR)	4 (0.5–7.5)	3 (1–5)	
Ulceration			0.060^a^
Absent	98 (61.3)	2047 (69.5)	
Present	49 (30.6)	730 (24.8)	
Missing	13 (8.1)	168 (5.7)	
Tumor type			< 0.001^c^
Superficial spreading melanoma	39 (24.4)	1264 (42.9)	
Nodular melanoma	64 (40.0)	935 (31.7)	
Acral lentiginous melanoma	3 (1.9)	48 (1.6)	
Lentigo maligna melanoma	13 (8.1)	49 (1.7)	
Desmoplastic melanoma	22 (13.8)	268 (9.1)	
Other	0 (0)	12 (0.4)	
Missing	19 (11.9)	369 (12.5)	
Clark level			< 0.001^c^
II	5 (3.1)	49 (1.7)	
III	32 (20.0)	784 (26.6)	
IV	87 (54.4)	1847 (62.7)	
V	28 (17.5)	223 (7.6)	
Missing	8 (5.0)	42 (1.4)	
No. of drainage sites			0.004^c^
0	1 (0.6)	0 (0.0)	
1	110 (68.8)	2281 (77.5)	
2	46 (28.7)	565 (19.2)	
3	3 (1.9)	82 (2.8)	
4	0 (0)	14 (0.5)	
Missing	0 (0)	3 (0.1)	
Drainage site of identified SNs			< 0.001^c^
Axilla	69 (43.1)	1453 (49.3)	
Groin	20 (12.5)	789 (26.8)	
Neck	62 (38.8)	618 (21.0)	
Popliteal	1 (0.6)	16 (0.5)	
Other	7 (4.4)	66 (2.2)	
Missing	1 (0.6)	3 (0.1)	
No. of SNs identified on lymphoscintigram			0.04^c^
0	1 (0.6)	1 (0)	
1	34 (21.3)	809 (27.5)	
2	56 (35.0)	984 (33.4)	
≥ 3	69 (43.1)	1131 (38.4)	
Missing	0 (0)	20 (0.7)	

Data are expressed as *n* (%) unless otherwise specified

*SNB* sentinel node biopsy, *IQR* interquartile range, *SNs* sentinel nodes

^a^Pearson’s Chi-square

^b^Mann–Whitney *U* test

^c^Fisher’s exact test

### Survival

**Fig. 1:**
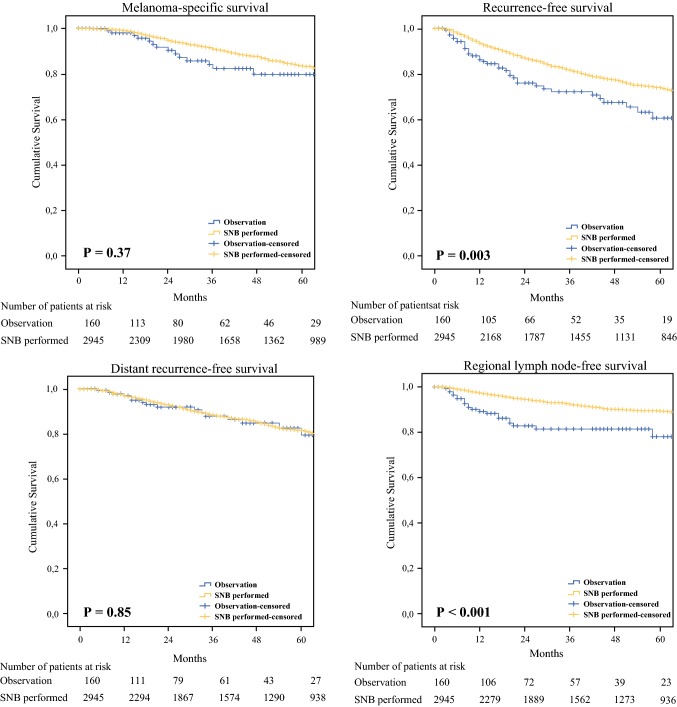
Melanoma-specific, recurrence-free, distant recurrence-free and regional lymph node-free survival according to type of management. *SNB* sentinel node biopsy

The median follow-up duration was 42 months (interquartile range 15–96 months). Of the 160 observed patients, 150 (94%) were followed with high-resolution US of their SNs at each follow-up visit. Of the remaining 10 patients, four were followed with only periodic physical examination of their lymph node fields, and six were lost to follow-up. The site of first recurrence differed between the observed and SNB patients (*p* = 0.03), with regional nodal recurrence being more common in the observed group (11% vs. 4%), while distant metastasis was more frequently seen in the SNB group (6% vs. 4%) (Table 2). SNB patients had significantly better RFS and RLNFS on univariable analysis (Table 3). MSS and DRFS were similar in the two groups (Fig. 1). After adjusting for all major prognostic factors, the multivariable analyses showed a superior RLNFS [observation group hazard ratio (HR) 2.0, 95% confidence interval (CI) 1.2–3.3]. MSS (observation group HR 0.9, 95% CI 0.6–1.6), RFS (observation group HR 1.1, 95% CI 0.7–1.5), and DRFS (observation group HR 0.9, 95% CI 0.5–1.5) were not significantly different between the two groups (Appendix Tables 4, 5).

**Table 2:** Characteristics regarding recurrence and treatment of patients

Characteristic	Observation (*n* = 160)	SNB (*n* = 2945)	*p* value
SN status			
Negative	NA	2531 (85.9)	NA
Positive	NA	404 (13.7)	
Missing	NA	10 (0.3)	
CLND			
Performed	NA	316 (10.7)	NA
Not performed	NA	2629 (89.3)	
Site of first recurrence			0.03^a^
Local	7 (4.4)	103 (3.5)	
In-transit	3 (1.9)	94 (3.2)	
Regional nodal	17 (10.6)	131 (4.4)	
Distant	6 (3.8)	163 (5.5)	
Multiple sites	4 (2.5)	110 (3.7)	
No. of metastatic nodes			
Mean (SD)	2.9 (2.7)	1.7 (1.7)	0.02^b^

Data are expressed as *n* (%) unless otherwise specified

*NA* not applicable, *SD* standard deviation, *SNB* sentinel node biopsy, *CLND* completion lymph node dissection

^a^Fisher’s exact test

^b^Mann–Whitney *U* test

**Table 3:** Results of univariable survival analysis

Variable	5-Year melanoma-specific survival	5-Year recurrence-free survival	5-Year regional lymph node-free survival	5-Year distant recurrence-free survival
Management				
Observation (%)	80	61	79	79
SNB (%)	84	74	90	82
*p* value	0.37	0.003	<0.001	0.85

**Table 4:** Cox multivariable analysis of melanoma-specific and recurrence-free survival

Factor	Value	Melanoma-specific survival			Recurrence-free survival		
		HR	95% CI	*p* value	HR	95% CI	*p* value
Management^a^	Observation	0.94	0.56–1.60	0.83	1.07	0.74–1.54	0.73
Sex	Male	1.42	1.12–1.80	0.004	1.25	1.05–1.49	0.01
Age	Per year	1.01	0.997–1.01	0.20	1.01	1.00–1.01	0.004
Melanoma location (reference: head and neck)	Upper limb	0.72	0.52–0.99	0.045	0.81	0.64–1.03	0.09
	Lower limb	0.84	0.61–1.15	0.27	0.98	0.78–1.25	0.89
	Trunk	0.94	0.70–1.14	0.65	0.72	0.57–0.91	0.005
Breslow thickness, mm (reference: 0–1 mm)	1.01–2	1.66	0.88–3.12	0.12	1.42	0.95–2.13	0.09
	2.01–4	3.14	1.67–5.89	< 0.001	2.46	1.63–3.69	< 0.001
	> 4	5.38	2.83–10.24	< 0.001	3.69	2.42–5.64	< 0.001
Tumor mitotic rate (reference: 0)	≥ 1	2.91	1.36–6.22	0.006	1.98	1.26–3.13	0.003
Ulceration	Present	1.71	1.37–2.13	< 0.001	1.62	1.36–1.92	< 0.001

*HR* hazard ratio, *CI* confidence interval

^a^Sentinel node biopsy versus observation

**Table 5:** Cox multivariable analysis of regional lymph node-free survival and distant recurrence-free survival

Factor	Value	Regional lymph node-free survival			Distant recurrence-free survival		
		HR	95% CI	*p* value	HR	95% CI	*p* value
Management^a^	Observation	1.99	1.21–3.29	0.007	0.88	0.53–1.46	0.61
Sex	Male	1.59	1.19–2.13	0.002	1.31	1.06–1.62	0.01
Age	Per year	1.01	1.00–1.02	0.09	1.00	0.99–1.01	0.67
Melanoma location (reference: head and neck)	Upper limb	1.20	0.79–1.80	0.39	0.70	0.52–0.94	0.02
	Lower limb	1.83	1.23–2.71	0.003	0.79	0.59–1.05	0.11
	Trunk	0.79	0.52–1.20	0.27	0.84	0.64–1.09	0.19
Breslow thickness, mm (reference: 0–1 mm)	1.01–2	1.93	0.92–4.03	0.08	1.59	0.95–2.67	0.08
	2.01–4	2.62	1.24–5.50	0.01	3.01	1.80–5.05	< 0.001
	> 4	2.99	1.38–6.48	0.006	4.56	2.67–7.79	< 0.001
Tumor mitotic rate (reference: 0)	≥ 1	5.00	1.59–15.77	0.006	1.84	1.07–3.17	0.03
Ulceration	Present	1.87	1.41–2.47	< 0.001	1.76	1.44–2.16	< 0.001

*HR* hazard ratio, *CI* confidence interval

^a^Sentinel node biopsy versus observation

### Immediate Versus Delayed Lymphadenectomy

Twenty-one patients (13%) developed a recurrence in a node field that was being observed. US detected these nodal recurrences in seven patients (33%), CT in three patients (14%), four patients (19%) were detected at physical examination by a doctor, and the remaining seven patients (33%) noticed the recurrence themselves. The nodal recurrence was directly underneath the tattoo in seven patients (33%). Two of the seven patients in whom the nodal recurrence was detected by US were found to have synchronous distant metastasis.

Fourteen of the 21 patients (66%) underwent therapeutic CLND. Limited local node excision with adjuvant radiotherapy was performed in one patient with cervical lymph node metastases. Widespread distant metastatic disease was the reason for not performing nodal surgery in two patients, two patients declined an operation, one patient died within 1 month after diagnosis of the regional nodal recurrence, and in one elderly patient with rapidly progressing disease and a recent deep venous thrombosis, surgery was considered inappropriate. The mean number of metastatic nodes in those patients who underwent therapeutic CLND was higher than in those patients who underwent immediate CLND because of an involved SN (2.9 vs. 1.7; *p* = 0.02).

## Discussion

Surgical decision making in elderly and frail patients is often complex and occasionally the risks of a staging procedure outweigh the benefits. Increasing incidence and mortality rates of elderly melanoma patients emphasize the importance of an adequate management strategy for this group of patients.^20^,^21^ The present study shows that focused US follow-up after lymphoscintigraphy proved to be an acceptable approach in elderly or frail patients in whom it has been decided to avoid SNB. It allows early diagnosis of nodal metastases, albeit not as early as with SNB, and does not jeopardize MSS, RFS or DRFS.

Previous research has demonstrated that SNB is readily able to be performed in the older population, and, in the majority of elderly patients, the SN is in fact procured.^7^,^22^–^26^ In our study, 75% of patients aged 75 years or older underwent the procedure, and it was still performed in 47% of those aged 85 years and over. The emergence of effective adjuvant systemic treatment in node-positive patients makes SNB an even more important staging tool, although the effectiveness of drug therapy in frail patients is currently uncertain since only patients with an Eastern Cooperative Oncology Group (ECOG) performance status < 2 were included in the trials that have been performed.^3^–^5^ Not performing SNB impedes access to adjuvant systemic therapy. Still, it is unclear whether adjuvant therapy improves MSS more than systemic therapy after a recurrence is detected. SNB is already therapeutic in a large proportion of node-positive patients.^1^,^12^

While SNB is an important staging tool, only 13% of the observed patients developed a regional nodal recurrence, and the other 87% would not have benefited from the procedure. Other reasons to be more restrained when considering SNB in the elderly population are the overall higher risk of operative and postoperative morbidity, the lower rate of nodal involvement, and the higher false negative rate of the procedure.^6^–^8^,^27^,^28^ Although SNB is a minor and fairly superficial procedure away from the vital organs and carries little morbidity, general anesthesia is typically used. 29,30 Performing SNB under local anesthesia may be technically feasible but this is not common practice in most centers.^31^

Some earlier studies have shown a correlation between comorbidity or performance status and the decision to perform SNB, while others have not.^22^,^25^,^26^ In our study, comorbid conditions were the reason for not performing the procedure in all patients < 65 years of age in whom SNB was omitted. A heterogeneous group of conditions was identified, varying from psychiatric ailments to bleeding disorders and cardiovascular conditions.

The current study is unique in that lymphoscintigraphy was performed in all patients, despite the fact that SNB was intentionally not scheduled, followed by focused US of the identified lymph nodes at each visit. The exact location of the SNs was marked on the skin with a permanent tattoo spot, allowing accurate repeated assessment. High-resolution US was performed at each follow-up visit in 94% of the observed patients. Depending on the drainage region, US is able to pick up metastatic nodes that are two to three times smaller than can be detected by physical examination.^32^ For the majority of patients, focused US did not add to the follow-up in an impactful way in our study. Most regional lymph node metastases were not detected by focused US. In only one-third of patients was US able to identify nodal metastases before they became otherwise apparent.

Although observed patients were considered unfit for SNB, 66% of the observed patients with a regional nodal recurrence still underwent therapeutic CLND. Recently, we showed that excision of clinically positive metastatic cervical lymph nodes followed by radiotherapy is a reasonable alternative for therapeutic CLND in frail patients.^33^ This new approach was used for one of the three observed patients with cervical macrometastasis. As shown previously, observed patients who developed nodal macrometastasis and underwent regional node dissection had significantly more involved nodes compared with SN-positive patients who had CLND.^1^ Even though previous research has shown that survival correlates inversely with number of involved nodes, MSS of the observed and SNB groups did not differ significantly in the present study, possibly due to small numbers.^12^

There are several limitations affecting this study. For instance, ECOG performance status was not formally assessed and recorded for all patients. Other limitations were the retrospective design, selection bias, and short follow-up for some patients.

## Conclusions

Omission of SNB due to advanced age and/or comorbidities occurred in 5.2% of patients in whom the procedure would generally have been considered appropriate. In comparison with patients who underwent SNB, these patients were older and had more advanced melanomas that were more often located in the head and neck region. The MSS, RFS, and DRFS rates were similar in the two groups, while RLNFS was worse in observed patients. Lymphoscintigraphy with focused US follow-up of identified SNs thus appears to be a reasonable management strategy to avoid SNB in patients who are elderly or have substantial comorbidities.

## Appendix

See Tables 4 and 5.
